# 1,4-Bis(4*H*-1,2,4-triazol-4-yl)benzene dihydrate

**DOI:** 10.1107/S1600536812029273

**Published:** 2012-07-10

**Authors:** Xiu-Guang Wang, Jian-Hui Li, Bin Ding, Gui-Xiang Du

**Affiliations:** a Tianjin Key Laboratory of Structure and Performance for Functional Molecules, Tianjin Normal University, Tianjin 300071, People’s Republic of China; b Pingdingshan Education Institute, Henan 467000, People’s Republic of China

## Abstract

The asymmetric unit of the title compound, C_10_H_8_N_6_·2H_2_O, comprises half the organic species, the mol­ecule being completed by inversion symmetry, and one water mol­ecule. The dihedral angle between the 1,2,4-triazole ring and the central benzene ring is 32.2 (2)°. The water mol­ecules form O—H⋯N hydrogen bonds with N-atom acceptors of the triazole rings. C—H⋯N hydrogen bonds are also observed, giving a three-dimensional framework.

## Related literature

For the synthesis of the title compound, see: Wiley & Hart (1953 ▶). For a comprehensive review on 1,2,4-triazole and its derivatives, see: Haasnoot (2000 ▶). For iron(II) compounds containing 1,2,4-triazole ligands, see: Kahn *et al.* (1998 ▶). For 1,2,4-triazole and its derivatives, see: Aromí *et al.* (2011 ▶). For C—N bond lengths, see: Heyrovska (2008 ▶); Schoknecht & Kempe (2004 ▶).
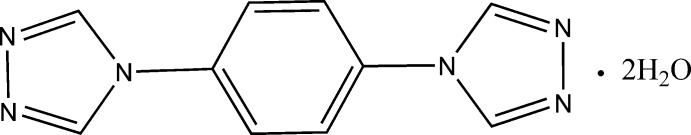


## Experimental

### 

#### Crystal data

C_10_H_8_N_6_·2H_2_O
*M* *_r_* = 248.26
Monoclinic, 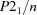

*a* = 3.7090 (7) Å
*b* = 15.680 (3) Å
*c* = 9.6054 (18) Å
β = 99.748 (3)°
*V* = 550.56 (18) Å^3^
*Z* = 2
Mo *K*α radiation
μ = 0.11 mm^−1^
*T* = 173 K
0.18 × 0.05 × 0.04 mm

#### Data collection

Bruker APEXII CCD diffractometer
Absorption correction: multi-scan (*SADABS*; Sheldrick, 1996 ▶) *T*_min_ = 0.993, *T*_max_ = 0.996
2717 measured reflections
959 independent reflections
838 reflections with *I* > 2σ(*I*)
*R*_int_ = 0.022

#### Refinement

*R*[*F* ^2^ > 2σ(*F* ^2^)] = 0.055
*wR*(*F* ^2^) = 0.129
*S* = 1.18
959 reflections
88 parameters
2 restraints
H atoms treated by a mixture of independent and constrained refinement
Δρ_max_ = 0.35 e Å^−3^
Δρ_min_ = −0.43 e Å^−3^

### 

Data collection: *APEX2* (Bruker, 2008 ▶); cell refinement: *SAINT* (Bruker, 2008 ▶); data reduction: *SAINT*; program(s) used to solve structure: *SHELXS97* (Sheldrick, 2008 ▶); program(s) used to refine structure: *SHELXL97* (Sheldrick, 2008 ▶); molecular graphics: *SHELXTL* (Sheldrick, 2008 ▶); software used to prepare material for publication: *publCIF* (Westrip, 2010 ▶) and *PLATON* (Spek, 2009 ▶).

## Supplementary Material

Crystal structure: contains datablock(s) global, I. DOI: 10.1107/S1600536812029273/rn2105sup1.cif

Structure factors: contains datablock(s) I. DOI: 10.1107/S1600536812029273/rn2105Isup2.hkl

Supplementary material file. DOI: 10.1107/S1600536812029273/rn2105Isup3.cml

Additional supplementary materials:  crystallographic information; 3D view; checkCIF report

## Figures and Tables

**Table 1:** Hydrogen-bond geometry (Å, °)

*D*—H⋯*A*	*D*—H	H⋯*A*	*D*⋯*A*	*D*—H⋯*A*
C1—H1⋯N3^i^	0.95	2.39	3.307 (3)	163
O1—H134⋯N2	0.88 (2)	1.98 (4)	2.816 (5)	158 (10)

Symmetry code: (i) 
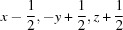
.

**Fig. 1.**
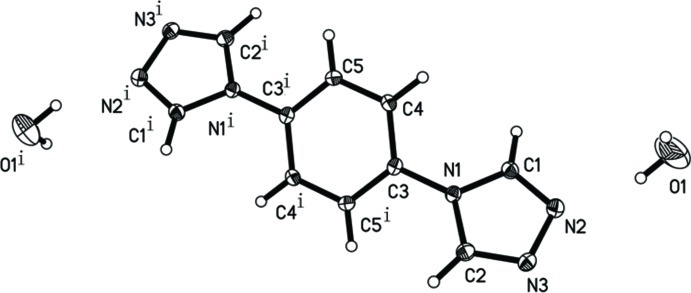
The molecular structure of (I), showing 30% probability displacement ellipsoids and the atom-numbering schemes. The superscript (I) on the atom labels corresponds to atoms generated by the symmetry operation: -x, -y, 1-z.

**Fig. 2.**
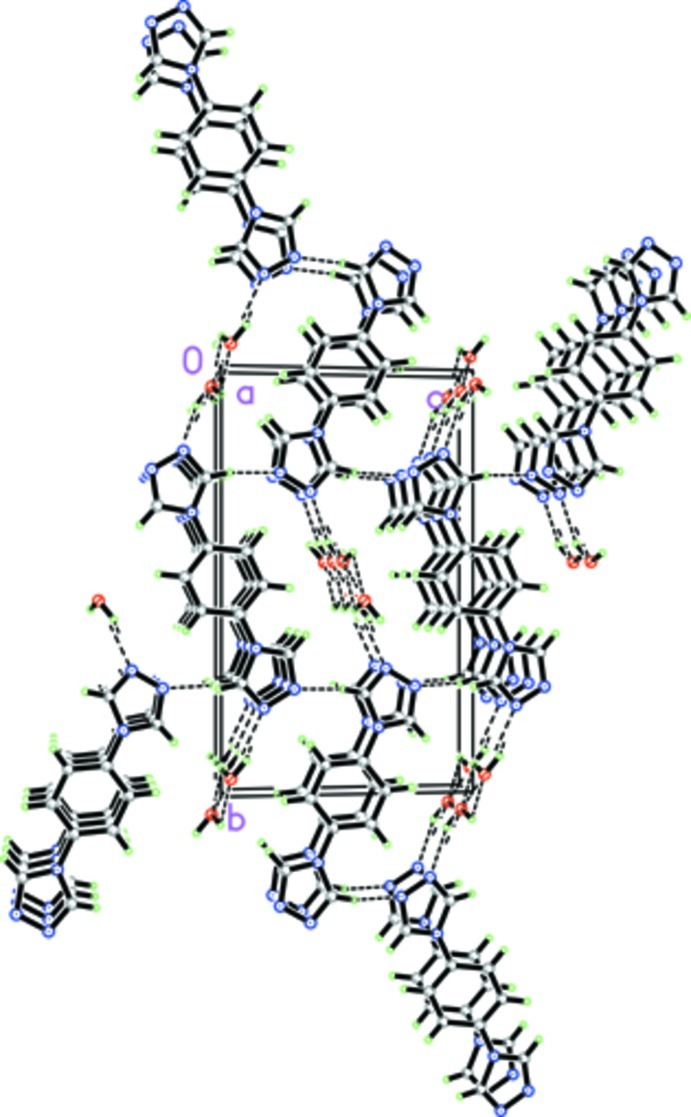
A three-dimensional supramolecular sheet structure of the title compound. The crystal packing for (I), with the H bond interactions shown as dashed lines.

## supplementary crystallographic information

### Comment

Nowadays 1,2,4-triazole and its derivatives have attracted great interest because they combine the coordination modes of pyrazole and imidazole. Some iron(II) complexes containing 1,2,4-triazole ligands have spin-crossover properties, which could be used in molecular-based memory devices, displays and optical switches. (Kahn *et al.*, 1998).

A comprehensive review about 1,2,4-triazole and its derivatives also has been made by Haasnoot (Haasnoot, 2000). One of the requirements for producing such macroscopic properties is to create interactions between the molecular units and the active sites within the crystal lattices. 1,2,4-Triazole and in particular its derivatives are very interesting as bridging ligands (Aromí *et al.*, 2011).

Herein we report the synthesis and the crystal structure of the title compound, 1,4-bis(4*H*-1,2,4- triazol-4-yl)benzene dihydrate, (I), which is expected to act as a tetradentate ligand to form low and multi-dimensional polymers with metal atoms.

The molecular structure of (I) is shown in Fig. 1. The triazole ring is almost perfectly planar maximum deviation from the least-squares plane is 0.001 (2) Å]. The distribution of bond lengths in the triazole ring vary from 1.305 (1)-1.430 (3) Å, which all fall in the range between 1.47 Å for a C-N single bond and 1.29 Å for a C=N double bond. The dihedral angle between the substituted benzene ring and the 1,2,4-triazole ring is 32.2 (2)°. The C1-N2 bond distance is 1.305 (1) Å and the C3-N1 bond distance is 1.430 (3) Å (Schoknecht & Kempe, 2004; Heyrovska, 2008), indicating that they correspond to double and single bonds, respectively. The C1-N1 bond distance is 1.352 (3) Å, which is 0.078 Å shorter than C3-N1 and 0.047 Å longer than C1-N2, indicating that there is a electron delocalisation in the 1,2,4-triazole π system.

Examination of the crystal structure with *PLATON* (Spek, 2009) shows the water molecules form O-H···N hydrogen bonds with the N-atom acceptors of the triazole rings, O-H···N and C1-H···N3 hydrogen bonds assemble (I) into a three dimensional supramolecular structure (Table 1, Figure 2) .

### Experimental

The title compound was obtained by the reaction of *N*,*N*'-diformylhydrazine (880 mg, 10 mmol) and *p*-pheneylenediamine (540 mg, 5 mmol) in the presence of ammonium sulfate (264 mg, 2 mmol) for 5 h at 413 K (Wiley & Hart,1953). The title compound were collected and recrystallized from methanol. After several recrystallizations from methanol, the air-dried product was obtained as colorless block crystals. Anal. Calcd for C_10_H_12_N_6_O_2_: C, 48.38; H, 4.87%. Found: C, 48.85; H, 4.95%..

### Refinement

The water H atoms were located in a Fourier difference map and refined subject to an O-H restraint 0.88 (1) Å and an H···H restraint of 1.42 (2) Å. Other H atoms were allowed to ride on their parent atoms with C-H distances of 0.93 Å and *U*_iso_(H) = 1.2Ueq(C). All of the non-hydrogen atoms were refined anisotropically.

### Crystal data

**Table:**

C_10_H_8_N_6_·2H_2_O	*F*(000) = 260
*M**_r_* = 248.26	*D*_x_ = 1.497 Mg m^−^^3^
Monoclinic, *P*2_1_/*n*	Mo *K*α radiation, λ = 0.71073 Å
Hall symbol: -P 2yn	Cell parameters from 2717 reflections
*a* = 3.7090 (7) Å	θ = 4.3–25.0°
*b* = 15.680 (3) Å	µ = 0.11 mm^−^^1^
*c* = 9.6054 (18) Å	*T* = 173 K
β = 99.748 (3)°	Block, colorless
*V* = 550.56 (18) Å^3^	0.18 × 0.05 × 0.04 mm
*Z* = 2	

### Data collection

**Table:**

Bruker APEXII CCD diffractometer	959 independent reflections
Radiation source: fine-focus sealed tube	838 reflections with *I* > 2σ(*I*)
Graphite monochromator	*R*_int_ = 0.022
φ and ω scans	θ_max_ = 25.0°, θ_min_ = 4.3°
Absorption correction: multi-scan (*SADABS*; Sheldrick, 1996)	*h* = −2→4
*T*_min_ = 0.993, *T*_max_ = 0.996	*k* = −18→18
2717 measured reflections	*l* = −11→11

### Refinement

**Table:**

Refinement on *F*^2^	2 restraints
Least-squares matrix: full	H atoms treated by a mixture of independent and constrained refinement
*R*[*F*^2^ > 2σ(*F*^2^)] = 0.055	*w* = 1/[σ^2^(*F*_o_^2^) + (0.0241*P*)^2^ + 0.9002*P*] where *P* = (*F*_o_^2^ + 2*F*_c_^2^)/3
*wR*(*F*^2^) = 0.129	(Δ/σ)_max_ < 0.001
*S* = 1.18	Δρ_max_ = 0.35 e Å^−^^3^
959 reflections	Δρ_min_ = −0.43 e Å^−^^3^
88 parameters	

### Special details

**Table:**

Geometry. All e.s.d.'s (except the e.s.d. in the dihedral angle between two l.s. planes) are estimated using the full covariance matrix. The cell e.s.d.'s are taken into account individually in the estimation of e.s.d.'s in distances, angles and torsion angles; correlations between e.s.d.'s in cell parameters are only used when they are defined by crystal symmetry. An approximate (isotropic) treatment of cell e.s.d.'s is used for estimating e.s.d.'s involving l.s. planes.
Refinement. Refinement of *F*^2^ against ALL reflections. The weighted *R*-factor *wR* and goodness of fit *S* are based on *F*^2^, conventional *R*-factors *R* are based on *F*, with *F* set to zero for negative *F*^2^. The threshold expression of *F*^2^ > σ(*F*^2^) is used only for calculating *R*-factors(gt) *etc*. and is not relevant to the choice of reflections for refinement. *R*-factors based on *F*^2^ are statistically about twice as large as those based on *F*, and *R*- factors based on ALL data will be even larger.

### Fractional atomic coordinates and isotropic or equivalent isotropic displacement parameters (Å^2^)

**Table:**

	*x*	*y*	*z*	*U*_iso_*/*U*_eq_	
N1	0.1379 (5)	0.15524 (13)	0.3739 (2)	0.0198 (5)	
N2	0.2094 (6)	0.29226 (14)	0.3416 (2)	0.0262 (6)	
N3	0.2767 (6)	0.24992 (14)	0.2222 (2)	0.0251 (6)	
C1	0.1282 (7)	0.23438 (16)	0.4290 (3)	0.0232 (6)	
H1	0.0699	0.2463	0.5195	0.028*	
C2	0.2311 (7)	0.16877 (17)	0.2441 (3)	0.0248 (6)	
H2	0.2590	0.1249	0.1785	0.030*	
C3	0.0687 (6)	0.07598 (15)	0.4383 (3)	0.0194 (6)	
C4	0.1572 (7)	0.06740 (16)	0.5842 (3)	0.0207 (6)	
H4	0.2642	0.1136	0.6407	0.025*	
C5	0.0875 (7)	−0.00908 (16)	0.6460 (3)	0.0210 (6)	
H5	0.1460	−0.0159	0.7455	0.025*	
O1	0.229 (3)	0.4543 (3)	0.4689 (5)	0.181 (3)	
H134	0.19 (3)	0.411 (4)	0.410 (8)	0.235*	
H13	0.448 (12)	0.434 (6)	0.498 (10)	0.181*	

### Atomic displacement parameters (Å^2^)

**Table:**

	*U*^11^	*U*^22^	*U*^33^	*U*^12^	*U*^13^	*U*^23^
N1	0.0191 (11)	0.0195 (11)	0.0213 (11)	−0.0001 (8)	0.0050 (8)	0.0013 (9)
N2	0.0275 (12)	0.0236 (12)	0.0276 (12)	0.0004 (9)	0.0048 (9)	0.0008 (9)
N3	0.0255 (12)	0.0253 (12)	0.0249 (12)	−0.0015 (9)	0.0057 (9)	0.0039 (9)
C1	0.0231 (14)	0.0227 (13)	0.0236 (13)	0.0012 (10)	0.0038 (10)	−0.0021 (11)
C2	0.0241 (14)	0.0266 (14)	0.0240 (14)	−0.0001 (11)	0.0052 (10)	0.0013 (11)
C3	0.0164 (12)	0.0191 (12)	0.0235 (13)	0.0006 (10)	0.0058 (10)	0.0024 (10)
C4	0.0198 (13)	0.0190 (12)	0.0228 (13)	0.0000 (10)	0.0023 (10)	−0.0029 (10)
C5	0.0216 (13)	0.0233 (13)	0.0181 (12)	0.0007 (10)	0.0031 (10)	0.0007 (10)
O1	0.365 (10)	0.067 (3)	0.089 (3)	0.028 (4)	−0.023 (4)	−0.036 (2)

### Geometric parameters (Å, º)

**Table:**

N1—C1	1.352 (3)	C3—C4	1.391 (3)
N1—C2	1.366 (3)	C4—C5	1.382 (4)
N1—C3	1.430 (3)	C4—H4	0.9500
N2—C1	1.305 (3)	C5—C3^i^	1.390 (4)
N2—N3	1.385 (3)	C5—H5	0.9500
N3—C2	1.305 (3)	O1—H134	0.88 (2)
C1—H1	0.9500	O1—H134	0.88 (2)
C2—H2	0.9500	O1—H13	0.87 (2)
C3—C5^i^	1.390 (4)		
			
C1—N1—C2	104.0 (2)	C5^i^—C3—N1	119.4 (2)
C1—N1—C3	127.6 (2)	C4—C3—N1	119.4 (2)
C2—N1—C3	128.4 (2)	C5—C4—C3	119.2 (2)
C1—N2—N3	107.0 (2)	C5—C4—H4	120.4
C2—N3—N2	106.7 (2)	C3—C4—H4	120.4
N2—C1—N1	111.3 (2)	C4—C5—C3^i^	119.6 (2)
N2—C1—H1	124.4	C4—C5—H5	120.2
N1—C1—H1	124.4	C3^i^—C5—H5	120.2
N3—C2—N1	111.0 (2)	H134—O1—H134	0 (10)
N3—C2—H2	124.5	H134—O1—H13	88 (8)
N1—C2—H2	124.5	H134—O1—H13	88 (8)
C5^i^—C3—C4	121.2 (2)		
			
C1—N2—N3—C2	0.2 (3)	C1—N1—C3—C5^i^	147.9 (3)
N3—N2—C1—N1	0.0 (3)	C2—N1—C3—C5^i^	−32.6 (4)
C2—N1—C1—N2	−0.2 (3)	C1—N1—C3—C4	−31.8 (4)
C3—N1—C1—N2	179.4 (2)	C2—N1—C3—C4	147.7 (3)
N2—N3—C2—N1	−0.3 (3)	C5^i^—C3—C4—C5	−0.1 (4)
C1—N1—C2—N3	0.3 (3)	N1—C3—C4—C5	179.6 (2)
C3—N1—C2—N3	−179.2 (2)	C3—C4—C5—C3^i^	0.1 (4)

Symmetry code: (i) −*x*, −*y*, −*z*+1.

### Hydrogen-bond geometry (Å, º)

**Table:**

*D*—H···*A*	*D*—H	H···*A*	*D*···*A*	*D*—H···*A*
C1—H1···N3^ii^	0.95	2.39	3.307 (3)	163
O1—H134···N2	0.88 (2)	1.98 (4)	2.816 (5)	158 (10)

Symmetry code: (ii) *x*−1/2, −*y*+1/2, *z*+1/2.

## References

[bb1] Aromí, G., Barrios, L. A., Roubeau, O. & Gamez, P. (2011). *Coord. Chem. Rev.* **255**, 485–546.

[bb2] Bruker (2008). *APEX2* and *SAINT* Bruker AXS Inc., Madison, Wisconsin, USA.

[bb3] Haasnoot, J. G. (2000). *Coord. Chem. Rev.* **131–135**, 200–248.

[bb4] Heyrovska, R. (2008). *Open Struct. Biol. J.* **2**,1–7.

[bb5] Kahn, O. & Martinez, C. J. (1998). *Science*, **279**, 44–48.

[bb6] Schoknecht, B. & Kempe, R. (2004). *Z. Anorg. Allg. Chem* **630**, 1377–1379.

[bb7] Sheldrick, G. M. (1996). *SADABS* University of Göttingen, Germany.

[bb8] Sheldrick, G. M. (2008). *Acta Cryst.* A**64**, 112–122. 10.1107/S0108767307043930 18156677

[bb9] Spek, A. L. (2009). *Acta Cryst.* D**65**, 148–155. 10.1107/S090744490804362X PMC2631630 19171970

[bb10] Westrip, S. P. (2010). *J. Appl. Cryst.* **43**, 920–925.

[bb11] Wiley, R. H. & Hart, A. J. (1953). *J. Org. Chem.* **18**, 1368–1371.

